# Inspiratory–Expiratory Muscle Training Improved Respiratory Muscle Strength in Dialysis Patients: A Pilot Randomised Trial

**DOI:** 10.3390/arm91010009

**Published:** 2023-02-10

**Authors:** Nicola Lamberti, Giovanni Piva, Yuri Battaglia, Michele Franchi, Matteo Pizzolato, Antonio Argentoni, Giorgio Gandolfi, Giulia Gozzi, Margherita Lembo, Pietro Lavisci, Alda Storari, Natascia Rinaldo, Fabio Manfredini, Annalisa Cogo

**Affiliations:** 1 Department of Neuroscience and Rehabilitation, University of Ferrara, Via Luigi Borsari 46, 44121 Ferrara, Italy; 2 PhD Program in Environmental Sustainability and Wellbeing, Department of Humanities, University of Ferrara, Via Paradiso 12, 44121 Ferrara, Italy; 3 Department of Medicine, University of Verona, Str. le Grazie, 8, 37134 Verona, Italy; 4 School of Sports and Exercise Medicine, University of Ferrara, Via Luigi Borsari 46, 44121 Ferrara, Italy; 5 Department of Medical Sciences, University of Ferrara, Via Aldo Moro 8, 44124 Ferrara, Italy; 6 Department of Rehabilitation Medicine, University Hospital of Ferrara, Via Aldo Moro 8, 44124 Ferrara, Italy

**Keywords:** exercise, rehabilitation, dialysis, respiratory muscles, strength, end-stage kidney disease, respiratory muscle training

## Abstract

**Highlights:**

**What are the main findings?**

- Low-intensity home-based breathing exercise improves respiratory muscle strength
- Only 4 weeks of training is required to improve maximal inspiratory–expiratory pressure

**What is the implication of the main finding?**

- Deconditioned dialysis patients may benefit from respiratory muscle training
- A home-based program autonomously executed is effective for preventing respiratory muscle function decline

**Abstract:**

End-stage kidney disease (ESKD) exposes patients to progressive physical deconditioning involving the respiratory muscles. The aim of this pilot randomized controlled trial was to determine the feasibility and effectiveness of low-intensity respiratory muscle training (RMT) learned at the hospital and performed at home. A group of ESKD patients (*n* = 22) were randomized into RMT or usual care (control group, CON) in a 1:1 ratio. The respiratory training was performed at home with an inspiratory–expiratory system for a total of 5 min of breathing exercises in a precise rhythm (8 breaths per minute) interspersed with 1 min of rest, two times per day on nondialysis days for a total of 4 weeks, with the air resistance progressively increasing. Outcome measures were carried out every 4 weeks for 3 consecutive months, with the training executed from the 5th to the 8th week. Primary outcomes were maximal inspiratory and expiratory pressure (MIP, MEP), while secondary outcomes were lung capacity (FEV1, FVC, MVV). Nineteen patients without baseline between-group differences completed the trial (T: *n* = 10; Age: 63 ± 10; Males: *n* = 12). Both MIP and MEP significantly improved at the end of training in the T group only, with a significant difference of MEP of 23 cmH_2_O in favor of the RMT group (*p* = 0.008). No significant variations were obtained for FVC, FEV1 or MVV in either group, but there was a greater decreasing trend over time for the CON group, particularly for FVC (t = −2.00; *p* = 0.046). Low-fatiguing home-based RMT, with a simple device involving both inspiratory and expiratory muscles, may significantly increase respiratory muscle strength.

## 1. Introduction

Chronic kidney disease (CKD) is responsible for a progressive alteration affecting skeletal muscles with a multifactorial etiology, including myocellular, immunologic and hormonal changes, metabolic acidosis, reduced protein intake and physical inactivity [1,2].

This condition, known as uremic myopathy, affects approximately 50% of patients on dialysis [3] and may lead to the development of sarcopenia [1], with impacts also on respiratory muscles.

Uremic myopathy is favorably counteracted by physical activity, both aerobic and anaerobic, which has been proven to be safe in patients on hemodialysis [2]. We previously observed in end-stage kidney disease (ESKD) patients that a home-based low-intensity walking training program, in addition to improving exercise capacity [4] and leg muscle strength, was also effective in counteracting the progressive deterioration of respiratory function and respiratory muscle strength [5].

However, to fight the decline in respiratory function, several training protocols specific for respiratory muscles have been developed [2], but there is still no consensus about optimal inspiratory muscle training (IMT) prescription regarding the duration of the intervention (ranging from 6 weeks to 6 months), number of times per day, numbers of sets and repetitions, devices and daily exercise load [2,6,7,8,9,10]. Despite large variability, past studies demonstrated the effectiveness of respiratory muscle training (RMT), even though the training protocols were often complex in terms of feasibility due to long training time, difficulty in device usage, and the need for direct supervision during exercise [2].

The aim of this pilot randomised controlled trial was to determine the feasibility and effectiveness of home-based low-intensity RMT on respiratory muscle strength and lung function using a positive pressure and volume exerciser device in ESKD patients.

## 2. Materials and Methods

This pilot randomized controlled clinical trial was conducted in the Operative Unit of Hemodialysis at Ferrara University Hospital in Ferrara, Italy. The study was approved by the Area-Vasta Emilia-Romagna Centro Ethics Committee with approval number 48/2019, and all participants provided written informed consent. The trial is reported following Consolidated Standards of Reporting Trials guidelines [11].

### 2.1. Subjects

The inclusion criteria were as follows: ESKD patients undergoing hemodialysis two to four times per week; males and females aged ≥18 years; and ability to walk for at least 10 m. The exclusion criteria were age under 18 years; impossibility of accomplishing the intervention; major lower-limb amputations; severe cognitive impairment; clinical condition contraindicating exercise; uncorrected anemia ([Hb] < 9 g/dL); and active infective disease (C-reactive protein > 10 mg/dL).

### 2.2. Randomization and Blinding

After the baseline data were collected, the participants were randomly assigned to receive RMT or usual care (control group, CON) with a 1:1 ratio randomization scheme. The outcome assessors were blinded to the patient allocation. Patients were not allowed to change groups for any reason.

### 2.3. Training Protocol

Respiratory muscle training was administered with the aid of an inspiratory expiratory system (Resp-in-out, Medinet, Milan, Italy, Supplementary Material Figure S1). This device consists of a positive pressure and volume exerciser for respiratory training, promoting inhalation and deep exhalation without interruption of the respiratory cycle. The tool is composed of a body into which the air is blown, and which contains a mobile ball; a tube with mouthpiece; and colored connectors with a grid filter, characterized by holes of different sizes (red, yellow and green), changing the resistance to airflow. The subjects, keeping their lips tightly close to the mouthpiece, exhale normally. The rising ball indicates the pressure in cmH_2_O. The pressure can increase according to the connector used. At the end of the exhalation, the ball goes down, and without detaching the lips from the mouthpiece, it is possible to complete the breathing exercise, making an inspiration. It is also possible to prolong inhalation and/or exhalation by keeping the ball in place, thus maintaining a certain pressure or a certain volume.

Patients were requested to perform the training on non-dialysis days (at least 4 days per week) two times per day, as follows. Using a metronome set at 30 beats per minute to guide the respiratory rhythm, patients were instructed to perform a complete respiratory cycle with inspiration synchronized with the first beat and expiration corresponding to the second beat and then wait for two consecutive beats before restarting. In one minute, patients performed 8 complete respiratory cycles. Then, the following minute, they rested without doing any training. This 1:1 min train-rest ratio was repeated five times for a total of 10 min. The training device included three different levels of resistance to the air flow, low, medium or hard, according to the dimensions and number of holes present in the connection between the mouthpiece and the device with the ball. During the first week of training, the air resistance was kept at a minimum; then, in week two, it was increased to the intermediate level, and finally, for the last two weeks, it was moved to the hardest level.

During the first week of exercise, an exercise physiologist supervised the training sessions of patients by directly administering them before the dialysis session. The following three weeks were performed at home autonomously by each patient. A daily log was provided to each patient to report the training execution and possible symptoms. Moreover, once per week on a dialysis day, a team member checked the training execution and reinforced the adherence to the program. At the end of the training period, to monitor the long-term adherence, patients of RMT group were allowed to keep the RMT device, but no recommendations were given to the patients in relation to the training continuation. 

### 2.4. Outcome Measures

The outcome measures were evaluated at four time points: baseline (T0); one month after baseline but before intervention (T1) to gauge a possible worsening of conditions; at the end of treatment (T2, after completing four weeks of training); and at the follow-up (T3, four weeks after the end of the training program). Outcome measures were collected before hemodialysis sessions.

The primary outcome measures were maximal inspiratory pressure (MIP) and maximal expiratory pressure (MEP), which were assessed by a vacuometer (MicroRPM, MD Spiro, Maine, US). Every test was performed three consecutive times, with a fixed resting period of 30 s within each trial, and the higher value was chosen. If the patients needed more time to recover, a longer suitable resting pause was allowed.

The secondary outcome measures included the following: forced expiratory volume at first second (FEV1), forced vital capacity (FVC) and maximal voluntary ventilation (MVV) measured by the Spiropalm 6MWT (Cosmed, Rome, Italy). Concerning the FEV1 and FVC, three trials separated by a 1 min resting period were performed; conversely, MVV was only tested once.

Each patient was familiarized with the outcome measures before performing the trials considered as outcomes. All the procedures were conducted according to the international guidelines [12,13].

Exercise capacity was assessed by a six-minute walk test carried out in a 20 m corridor, with each patient instructed to cover as much distance as possible in the given time [14].

### 2.5. Statistical Analysis

Standard methods of analyses for randomised controlled trials were employed. Data distribution was verified via the Shapiro-Wilk test. Continuous variables are reported as the mean ± standard deviation or mean (95% confidence interval), while categorical variables are reported as the median (interquartile range).

Baseline comparisons between groups were performed with independent samples *t* tests, Mann-Whitney U tests or Fisher’s exact test according to the nature and distribution of the data.

Within-group comparisons throughout each period were performed with paired samples *t* tests or Wilcoxon tests, as appropriate. Between-group comparisons of variations between T1 and T2 were carried out with independent samples *t* tests or Mann-Whitney tests. A repeated measure analysis of variance was performed to determine the trend of variation for the secondary outcome measures.

**Figure 1:**
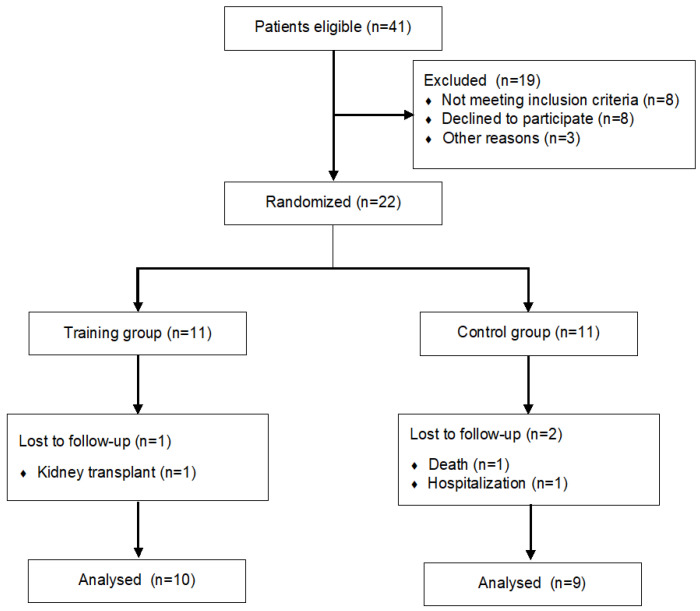
Study flow diagram.

Missing data, despite being present in a negligible number (*n* = 6) randomly placed, were replaced through multiple imputation. An intention-to-treat analysis was scheduled, but since 3 patients dropped out early in the trial (Figure 1), a sensitivity analysis was preferred to guarantee data sensitivity.

A *p* value < 0.05 was considered significant. Statistical analyses were performed with MedCalc Statistical Software version 20.014 (MedCalc Software Ltd., Ostend, Belgium).

## 3. Results

Twenty-two patients at Kidney Disease Outcomes Quality Initiative stage V undergoing maintenance hemodialysis were proposed to participate in the study between September 2021 and June 2022 at the Unit of Nephrology and Dialysis of University Hospital of Ferrara. Eleven patients were randomised into the RMT group, whereas the remaining 11 acted as the CON group (C). At baseline, the patients did not differ for any demographic or clinical characteristics, or for any outcome measure (Table 1).

Three patients dropped out early from the trial due to death or hospitalisation or for kidney transplantation (Figure 1). The final analysis was performed on 10 patients for the RMT group and 9 for the C group. Additionally, this subsample of patients did not present any baseline difference.

### 3.1. Training Features

The 10 patients randomised into the RMT group performed a median 75% (interquartile range 50–100%) of training sessions, with respect to the prescribed sessions, between T1 and T2. No symptoms, difficulties or adverse events were reported by the patients of RMT group during the training execution. After the end of the training period (T2), 5 patients in the RMT group reported to having spontaneously maintained a regular respiratory training pattern also for the follow up weeks.

### 3.2. Data Stability

A paired-sample comparison for all study participants, performed to check outcome measures’ stability over time, did not exhibit significant differences from T0 to T1 in all study participants (*n* = 22); data are reported in Table 2.

### 3.3. Primary Outcome

**Figure 2:**
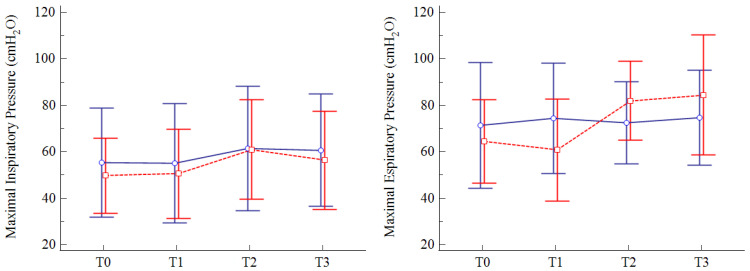
Distribution overtime for MIP and MEP values in the RMT group (red) and CON group (blue). Legend: data are reported as mean (95% confidence interval).

Both MIP and MEP values significantly improved in the RMT group at the end of training (T2), with MEP values that remained significantly higher than baseline at follow-up (T3). Stable values over time were observed for the CON group. A significant between-group difference in variations from baseline to the end of treatment was observed for MEP (*p* = 0.008), with a mean difference of 23 cmH_2_O (95% CI 7 to 39) in favor of the RMT group. No significant differences were noted for MIP variations (*p* = 0.53). The data are presented in Table 3 and Figure 2.

### 3.4. Secondary Outcomes

No significant variations were obtained for FVC, FEV1 or MVV in either group (Table 3). The trend of variation was stable for MVV for both the RMT group (t = +0.60; *p* = 0.56) and the CON group (t = −0.30; *p* = 0.76).

In the RMT group, slowly decreasing nonsignificant trends were observed for both FEV1 (t = −0.85; *p* = 0.37) and FVC (t = −0.65; *p* = 0.53). Otherwise, in the CON group, markedly decreasing trends were noted for FEV1 (t = −1.59; *p* = 0.15) and statistically significant for FVC (t = −2.00; *p* = 0.046).

No significant changes were recorded for the 6MWT at the different timepoints of collection.

### 3.5. Maintenance of Benefits and Adherence

As reported, 5 patients in the RMT group opted for maintaining the respiratory training schedule after the end of the training period (T2). This resulted in a maintenance of significantly different values for MEP with respect to T1 but not for MIP (Table 3).

### 3.6. Post-Hoc Power Calculation

A post hoc power calculation was performed for the MEP outcome. A combined power of 86.2% was obtained, considering the mean deviation from baseline at the end of treatment for the two groups, and assuming a type I error at 0.05.

## 4. Discussion

This pilot randomized controlled study aimed to test the ease of use and the effectiveness on the strength of respiratory muscles and on the effort-dependent spirometric values of a home-based novel training using a tool to exercise ventilation based not only on an increase in air flow resistance but also in volumes.

The trial was carried out in an ESKD population, where respiratory and peripheral muscle weakness is a systemic component, as reported in both in vivo and in vitro studies [15]. Indeed, muscle weakness affects exercise capacity, reducing participation in daily activities and the quality of life of these patients [16]. Respiratory muscle training as well as exercise training can counteract respiratory deterioration, improving lung function, fitness and quality of life [7,17,18,19,20] in CKD patients. Recently, the importance of lung function as a contributor to the overall exercise capacity in the general population has been further highlighted [21].

The first finding of the present pilot study is the possibility of significantly improving the strength of the respiratory muscles with a simple tool easily employed at home by patients. In fact, both MIP and MEP values significantly increased in the RMT group but not in CON. The increase observed in the RMT group was approximately 25 cmH_2_O, consistent with the values reported in other trials, but obtained in a shorter training period [4 weeks instead of at least 6-week periods] [2,6,7,8,9,10,22,23,24]. As a further intriguing observation, the persistence of benefits was observed at follow-up for the MEP value only, with the limitation that 50% of subjects maintained a quite regular training pattern. Scientific literature reported congruent variations in both MIP and MEP values [2,6,7,8,9,10,22,23,24] immediately after training, as we also observed, but to the best of our knowledge, no paper has compared the duration of the effect after the end of training in both parameters. Moreover, in previously published papers, more importance is given to MIP, and MEP is scarcely reported, probably because inhalation depends more on muscle strength than exhalation.

In relation to respiratory function, in the present pilot trial, no significant improvements were observed for the FVC, FEV1 and MVV parameters, in contrast with the papers of Campos et al. [22] and Yuenyongchaiwat et al. [23], where, however, a twofold longer training period was scheduled. This discrepancy may be related to the specificity of the training in relation to the muscle fiber adaptations induced in relation to the different fiber composition of respiratory muscles. Indeed, slow fibers and fast fibers are present in equal proportions in the adult human diaphragm while intercostal muscles contain a higher proportion of fast fibers [25]. As previously observed in trials [25], different training modalities (e.g., endurance versus resistance) contributed to selected muscle fiber adaptations in terms of cross-sectional area, oxidative enzymes, etc. In our study, the low–moderate intensity of the program may have contributed to an increase in the proportion of slow fibers and in the size of the fast fibers, especially in the intercostal muscles as previously reported also in COPD patients [25]. Otherwise, changes in lung function in dialysis patients are well described [3], particularly a progressive deterioration of vital capacity, probably due to respiratory muscle weakness. We also observed, in a previous paper, a progressive decline in respiratory function in ESKD patients that was counteracted by an exercise program [5]. In this trial, a decrease for both FEV1 and FVC was collected in a three-month observation period. However, in the RMT group, the decrease was approximately 50 mL, a value falling within the physiological variability accepted by the Guidelines (150 mL) [13], whereas a much greater, although not significant, decrease was observed in the CON group. These patients lost approximately 120 mL of FEV1 and 250 mL of FVC, with a significant decreasing trend.

The second important finding of the present study is the significant effectiveness of training even if of shorter duration and lower intensity with respect to all the other protocols observed in the literature. Indeed, in this pilot trial, only 80 breathing cycles per nondialysis day were required for the patients, divided into eight complete breaths against resistance, followed by one minute resting pauses, to be repeated five consecutive times, twice per day. Several differences from the published literature are reported in terms of training modality, duration and intensity. At first, in this trial, the training was executed at home autonomously by the patients, as in only two other trials [10,26], but in contrast with the papers of Dipp et al. and Medeiros et al., the exercise was performed only on the nondialysis day to avoid adding more fatigue to the ones reported by ESKD patients at the end of dialysis sessions [27]. Moreover, the respiratory training was conducted at a lower intensity than previously reported [2,6,7,8,9,10,22,23,24], also considering the intermittent characteristic of the exercise, which comprised one breathing cycle overcoming the resistance, interspersed by one quiet breathing cycle. Finally, the patients found the training feasible and safe, reporting no significant fatigue or adverse events; five patients also autonomously maintained the exercise pattern even after the end of the training period, underlying the feasibility of the training proposed. Nevertheless, the improvement of respiratory muscle strength, obtained in a short period and with low-intensity training, is a key point for the ability to ventilate larger volumes of air for a longer time with less fatigue, as required during exercise but also during the everyday activities of daily living in a deconditioned population, such as ESKD patients undergoing dialysis [27]. However, the problem of selecting the appropriate regimen for effective rehabilitation of the respiratory muscles is still an open issue and requires further research, especially in fragile populations as the ESKD patients.

The study has several limitations. First, the small sample size may have affected the significance of the results, as well the slight imbalances at baseline, particularly related to dialysis vintage. Quality of life was not directly measured in this pilot study. Moreover, the adherence of patients to the treatment was only reported on the daily diary, and was not confirmed by caregiver or monitored. Finally, patients of the RMT group decided autonomously whether to continue the training after the scheduled exercise period, potentially confounding the results observed at the follow-up.

## 5. Conclusions

In conclusion, the trial supported the hypothesis that low-intensity home-based RMT, with a simple device involving both inspiratory and expiratory muscles, may significantly increase respiratory muscle strength. These promising preliminary results need to be confirmed in a larger trial, when the minimum amount of training to maintain the benefits could also be established and when a comparison with other training protocol should be performed.

## Figures and Tables

**Table 1:** Demographic and clinical characteristics of the sample at baseline.

	Training (*n* = 11)	Control (*n* = 11)	*p*-Value
Age	62 ± 13	65 ± 11	0.60
Male sex	6	7	0.69
BMI	27.9 ± 5.0	26.2 ± 6.6	0.51
Years of dialysis	3 ± 2	4 ± 3	0.95
Smoking	6	7	0.69
Current Smoking	0	1	1.00
Hypertension	11	11	1.00
Hyperlipidemia	8	9	0.66
Diabetes	6	4	0.67
Charlson Index	5 ± 3	6 ± 3	0.23
Hemoglobin, g/dl	11.0 ± 1.7	11.5 ± 0.4	0.41
Serum creatinine, mg/dl	9.7 ± 3.0	9.4 ± 2.7	0.83
MIP (cmH_2_O)	48 ± 22	53 ± 28	0.65
MEP (cmH_2_O)	65 ± 24	69 ± 32	0.78
FEV1 (L)	2.22 ± 0.78	2.29 ± 0.52	0.80
FVC (L)	2.76 ± 0.94	3.05 ± 0.78	0.44
MVV (L)	69 ± 24	82 ± 26	0.22
6MWT (m)	315 ± 136	322 ± 69	0.80

**Table 2:** Comparison between outcome measures from T0 to T1 in the entire population (*n* = 22).

	T0	T1	*p*-Value
MIP (cmH_2_O)	52 (40–65)	53 (39–67)	0.92
MEP (cmH_2_O)	68 (53–82)	67 (52–82)	0.83
FEV1 (L)	2.24 (1.93–2.56)	2.19 (1.89–2.48)	0.19
FVC (L)	2.91 (2.49–3.34)	2.81 (2.45–3.17)	0.12
MVV (L)	73 (61–86)	73 (60–87)	0.90

Legend: Data are reported as mean (95% confidence interval).

**Table 3:** Outcome measures in both the training and control groups. * *p* < 0.05 respect to T0 measured with paired-samples test.

	RMT Group (*n* = 10)				CON Group (*n* = 9)			
	T0	T1	T2	T3	T0	T1	T2	T3
MIP (cmH_2_O)	50 (19–91)	51 (17–94)	61 * (30–112)	56 (19–115)	55 (16–111)	55 (14–113)	61 (16–124)	63 (16–113)
MEP (cmH_2_O)	64 (29–100)	61 (26–112)	82 * (55–128)	84 * (39–136)	71 (41–134)	74 (45–132)	72 (43–113)	75 (40–113)
FEV1 (L)	2.29 (1.20–3.47)	2.23 (1.12–3.19)	2.19 (1.12–3.28)	2.23 (1.20–3.18)	2.20 (1.46–2.89)	2.14 (1.47–2.85)	2.08 (1.40–1.74)	2.08 (1.33–1.58)
FEV1% predicted	80 (67–92)	79 (66–90)	76 (63–88)	79 (66–92)	82 (71–94)	79 (67–90)	78 (66–89)	79 (67–91)
FVC (L)	2.84 (1.73–4.39)	2.81 (1.73–4.01)	2.74 (1.73–4.06)	2.79 (1.73–3.85)	3.00 (1.99–4.52)	2.81 (1.88–3.95)	2.75 (1.77–3.92)	2.75 (1.73–2.85)
FVC% predicted	79 (69–90)	79 (70–88)	78 (70–86)	79 (68–89)	83 (73–93)	81 (71–91)	81 (63–96)	80 (64–96)
MVV (L)	68 (36–113)	67 (33–111)	68 (45–103)	73 (46–127)	79 (37–120)	79 (38–122)	82 (51–131)	77 (49–120)
6MWD (Meters)	306 (162–449)	296 (172–421)	327 (229–425)	-	322 (266–379)	297 (251–344)	307 (268–344)	-

## Data Availability

The dataset originated from the trial is available upon request to the corresponding author.

## Supplementary Materials

The following supporting information can be downloaded at: https://www.mdpi.com/article/10.3390/arm91010009/s1, Figure S1: The respiratory training device.
