# Supplementary figures and images for: Inspiratory–Expiratory Muscle Training Improved Respiratory Muscle Strength in Dialysis Patients: A Pilot Randomised Trial
Source: Adv Respir Med. 2023 Feb 10;91(1):93–102. doi: 10.3390/arm91010009 (PMC9952421; doi:10.3390/arm91010009)

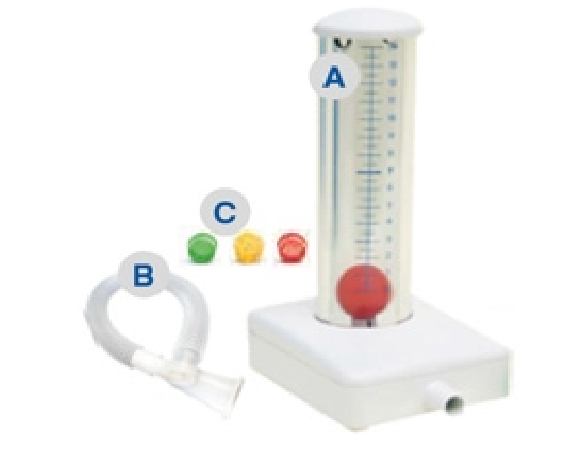

Supplement: Supplementary file 1 [file arm-91-00009-s001.zip › arm-2114103-supplementary.png]
